# The Neuro-Hormonal Control of Rapid Dynamic Skin Colour Change in an Amphibian during Amplexus

**DOI:** 10.1371/journal.pone.0114120

**Published:** 2014-12-03

**Authors:** Christina Kindermann, Edward J. Narayan, Jean-Marc Hero

**Affiliations:** Environmental Futures Research Institute, School of Environment, Griffith University, Gold Coast Campus, Southport, Australia; Arizona State University, United States of America

## Abstract

Sexual signalling using dynamic skin colouration is a key feature in some vertebrates; however, it is rarely studied in amphibians. Consequently, little is known about the hormonal basis of this interesting biological phenomenon for many species. Male stony creek frogs (*Litoria wilcoxii*) are known to change dorsal colouration from brown to lemon yellow within minutes. This striking change is faster then what has been seen most amphibians, and could therefore be under neuronal regulation, a factor that is rarely observed in amphibians. In this study, we observed colour changes in wild frogs during amplexus to determine the natural timing of colour change. We also investigated the hypothesis that colour change is mediated by either reproductive or neuro- hormones. This was achieved by injecting frogs with epinephrine, testosterone, saline solution (control 1) or sesame oil (control 2). A non-invasive approach was also used wherein hormones and controls were administered topically. Male frogs turned a vivid yellow within 5 minutes of initiation of amplexus and remained so for 3–5 hours before rapidly fading back to brown. Epinephrine-treated frogs showed a significant colour change from brown to yellow within 5 minutes, however, testosterone-treated frogs did not change colour. Our results provide evidence of the role neuronal regulation plays in colour change systems.

## Introduction

Dynamic colour change is a reversible change is skin colour or tone that involves the dispersion or aggregation of pigments within dermal chromatophores (colour cells) following stimulation by hormones [1]. Dynamic colour change is commonly associated with camouflage or thermoregulation [2]–[4], but it is also an important part of communication (e.g. courtship and displays of dominance) in many species [5], [6]. Bright colours displayed during breeding events could increase vulnerability to predation and temporary colour changes can resolve this problem as animals need only to display bright colours during mating interactions and can essentially ‘turn them off’ if they sense danger [6]. Several amphibians are known to change colour for breeding displays, ranging from slower, more seasonal changes, to rapid changes in colour during calling or amplexus [7], [8]. For example, *Rana Arvalis* develops blue colouration for several days during breeding [9], whereas the yellow colouration on *Bufo luetkenii* fades back to brown over several hours during amplexus [10]. Such differences in colour displays suggest that varying functions and mechanistic processes of dynamics colour change in amphibians[11].

A number of hormones play a role in either dispersing or aggregating pigment in the dorsal skin of amphibians. Simple darkening and lightening of skin tone is common in this group and is controlled by alpha-melanocyte stimulating hormone (α-MSH), which triggers the dispersion of melanin into the arms of the melanophores [12]–[14] covering other colour cell layers. Its antagonist, melanin-concentrating hormone, has the opposite effect and triggers the aggregation of pigment that leads to the lightening of skin colour [15], [16]. Stress hormones such as catecholamines and ACTH, a pre-curser to corticosterone, and reproductive hormones can have differing effects on pigment movement [17], [18]. Numerous studies on amphibians displaying breeding colouration have shown that such changes are driven by androgens. Testosterone injections increased yellow breeding colouration in male *Buergeria robusta* [19], while female *Hyperolius viridiflavus* and *Acris gryllus* implanted with testosterone pellets developed male-specific breeding colouration [20], [21].

Aggressive social interactions during mating can also activate stress-related endocrine and/or neuroendocrine pathways, which can lead to changes in skin colour [22]. Neuro-hormones, such as the catecholamines (epinephrine, norepinephrine and dopamine), which are produced during the ‘fight or flight’ stress response, could be responsible for activating rapid colour change through the sympathetic nervous system. Additionally, the elevated plasma epinephrine levels in *Bufo japonicus* that occur during amplexus were linked with triggering sperm release from spermatophores [23], [24]. Although this has not been explored in relation to nuptial colouration, catecholamines can directly stimulate chromatophores and the colour response, depending on species [25].

**Figure 1:**
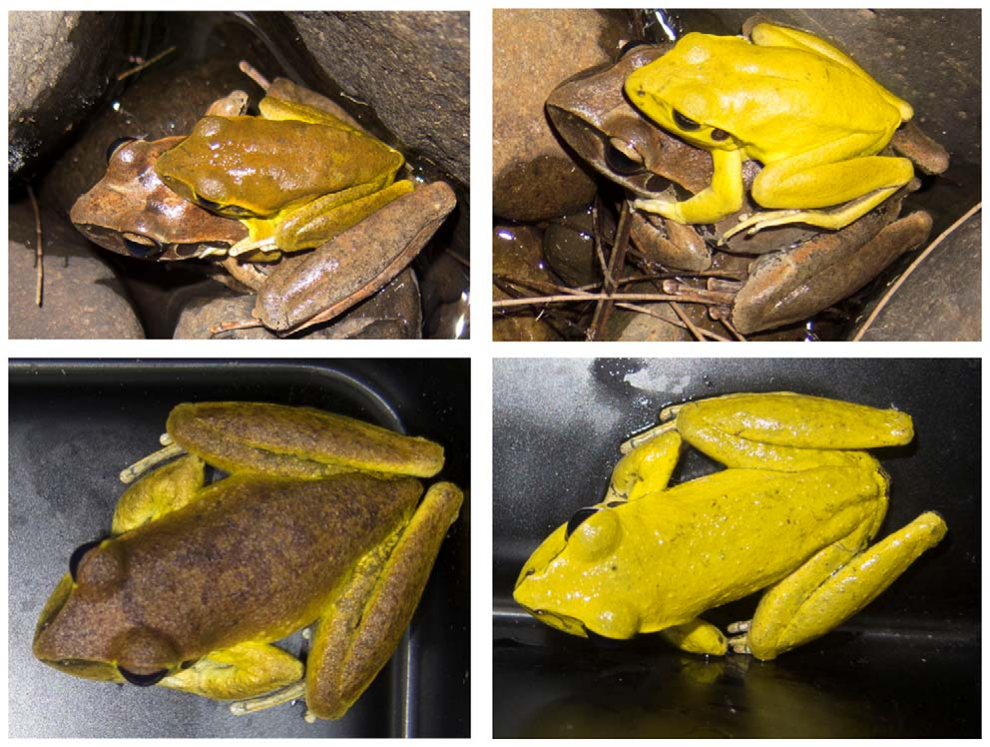
Male *L. wilcoxii* during amplexus at time 0 (top left), after 10 minutes (top right), baseline pre-amplectic male (bottom left), and post epinephrine injection (bottom right). Colour score for the brown male is −1.93 (RGB = 67, 58, 30) and the amplexing male is 3.01(RGB = 254, 249, 78).

Male *Litoria wilcoxii* [Fig 1] change colour during breeding events, most often during amplexus. In a previous study, it was determined that, minutes after application of a moderate stressor (toe-clipping), this species turned a bright lemon colour, similar to that of amplexing males [26]. We were intrigued by this phenomenon; in particular, we wished to determine whether this speed occurs naturally and to identify the hormonal mechanisms driving it. In the same study [26] we ruled out the role of the stress hormone corticosterone and its pre-curser ACTH which opened up the possibility of neuro-endocrine stress hormones such as adrenalin. The similarities between the colour change responses observed from toe-clipping and observations of wild males suggest that the same hormone is driving both responses and therefore indicates a possible neuro-hormone involvement [11], [27]. Yet we cannot rule out the role reproductive hormones may play, as the natural timing of this colour change is observed during breeding events. Identifying which of these hormones stimulates pigment movement would provide insight into the mechanistic processes of rapid colour change in amphibians.

In this study, we observed pairs prior to and during amplexus, using digital photography to document the natural timing of this striking colour change. We also conducted manipulations where male frogs were treated with epinephrine and testosterone to determine if this rapid colour response was associated with neuro-hormonal processes.

## Methods

### Ethics statement

This study was carried out under permit WISP13675913 issued by the Queensland Department of Environment and Heritage Protection (DEHP) and the Griffith University Animal Ethics Committee (AEC) approved the experiments (permit # ENV/20/12/AEC).

### General methods

This study was carried out in 2013 and 2014 in Numinbah Valley, South East Queensland, Australia (28.219°S, 153.232°E, and 196 m altitude). Aggregations of breeding males, consisting of approximately 30 individuals, were found along rocky creek sections of Nerang River. Natural observations were undertaken during the peak breeding season (October to January), with experiments occurring during the month of October.

### Dynamic colour change in male frogs during amplexus

Females encountered at the stream edge, where males are calling, were observed until amplexus was initiated. Following amplexus, the pairs (n = 19) were photographed at 0, 5, 10, 20 and 30 minutes and every subsequent 30-minutes until 1) the male frog changed back to brown, or 2) the pair could no longer be found.

### Experimental hormone treatments

After capture, male frogs were placed into black painted plastic boxes (30 cm×30 cm×20 cm) and photographed. Frogs (n = 5 individuals per treatment group) were, 1) injected with hormone (epinephrine or testosterone), 2) injected with a control solution (saline or oil), 3) exposed topically to epinephrine or testosterone, or 4) exposed topically to a control solution. Frogs were injected in the coelomic cavity, at the junction of the underbelly and thigh, away from the vital internal organs, using a thin 1 mL sterile syringe and a sterile 25-gauge needle. For topical treatments, the hormone or control solution was administered onto the dorsal surface of the frog using an eye dropper (same dose as injected frogs). Epinephrine (Sigma-Aldrich E4250) dosage for each frog was 10 µg/0.1 mL 0.9% NaCl (0.0055 M). The testosterone (Sigma-Aldrich T1500) dosage was 1 µg 0.1 mL pure sesame oil solution (0.30–10 M) [25]. The controls were 0.1 mL saline (C1) or 0.1 mL oil per frog (C2). Frogs were then photographed at 5, 10, 20, 30, 60 and 120 minutes. Between photographs, a plastic lid was placed on the box so that torch light could not impact skin colour change of the frog [28].

### Colour analysis and statistics

Colour analysis followed the methods described previously [26]. RAW format photographs were taken using a digital camera (Canon Powershot S5, Japan) with a sync macro setting and the flash at full. All frogs were placed next to A Munsell 24 Colour Checker Chart to demonstrate the similarity of exposure between photographs, and allow calibration if there were any differences [4], [29]. Photos were corrected for light differences in Adobe Photoshop C35 Extended [30] using the “white balance tool”. Following this, the photograph was cropped, leaving a rectangle showing only the dorsal body surface of the frog. Any pixels that were white as the result of flash reflection were removed using the “magic wand” tool. The average RGB (red, green blue) value for each edited rectangle was determined using the histogram function [31].

The data set (triplet values of R, G and B) were processed as basic analysis variables using correlations between variables by the PCA module in R [32]. Three axes (or factors) were computed (the major, semi-major and remaining variance), and the eigenvalues for each axis were 2.41, 0.52 and 0.04, which accounted for 81%, 17% and 1% of the variance. Because factors with eigenvalues less than 1 have less explanatory power than the original variables there is no statistical reason to retain them [33]. Thus the analysis results in a very satisfactory single factor which describes the changes in colour, which ranged from the darkest red to brightest yellow. To convert the RGB values for each data point to scores on the major axis, the standardised R, G and B values are multiplied by the factor loadings as provided by the PCA module in R. This step is automatically calculated using the phych package in R [32]. The loadings for factor (or axis 1) are: for R 0.392472, for G 0.399892 and for B 0.318050. The final values calculated ranged from −3 (dark brown) to +4 (lemon yellow) [Table S1].

All data were analysed in the statistical programming environment R [34]. Prior to analysis, we checked for normal distribution using the histogram function in R to ensure we met the normality assumption for the ANOVA model. Probability values of p<0. 05 were considered significant. RGB values are expressed as the mean (SD). To account for unequal sampling repetitions at each time point, colour change in amplexing frogs was analysed using linear mixed effect models and repeated measures. The response variable was colour. Time (levels ranged from 5 to 330 min) was a fixed effect, and the individual frog was a random effect. P-values were based on Markov-chain Monte Carlo sampling (MCMC) using the language R package, as the lme4 package does not give degrees of freedom, F statistics or p-values [35]. To test the significance of treatment and the differences between hormone/control groups in terms of increases in colour score (yellow colour), we used a two-way analysis of variance (ANOVA) with repeated measures on time, within a treatment group. Contrasts and interaction contrasts between the factors were computed using the multi-comp package [36].

## Results

### Dynamic colour change in male frogs during amplexus

**Figure 2:**
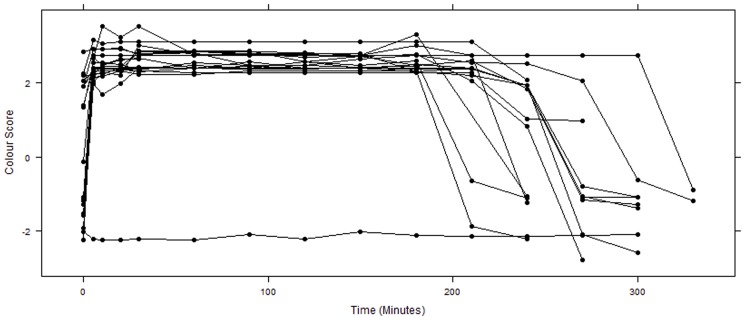
*L. wilcoxii* male dorsal colour change during amplexus (n = 19). Points indicate colour of each individual male, colour changes over time (0–300 minutes). Colour score of a single female throughout amplexus is provided as a reference (lower line where now increase is observed). There was a significant difference in colour between baseline and all tested time points (p = 0.0001) until 240 minutes (p = 0.2920). From time 270 to 330, scores were significantly lower than baseline (p = 0.0001).

The mean baseline (time 0) colour score for males at initiation of amplexus (time 0) was 0.32 (1.8). Frogs changed colour from brown to bright yellow (Fig's 1 and 2; 2.49(0.32) within 5 minutes and remained yellow until 240 minutes. By 330 minutes, males had faded back to brown, while remaining in amplexus (Fig 2; −1.02(0.21)). During amplexus, colour scores were significantly higher than baseline scores (Fig 2; p = 0.0001), with the exception of 240 minutes, when there was no significant difference (Fig 2; p = 0.2920) and 270, 300 and 330 minutes, when values were significantly lower than baseline (Fig 2; p = 0.0001), indicating that the frogs were darker than their original time 0 colour.

### Experimental hormone treatments

**Figure 3:**
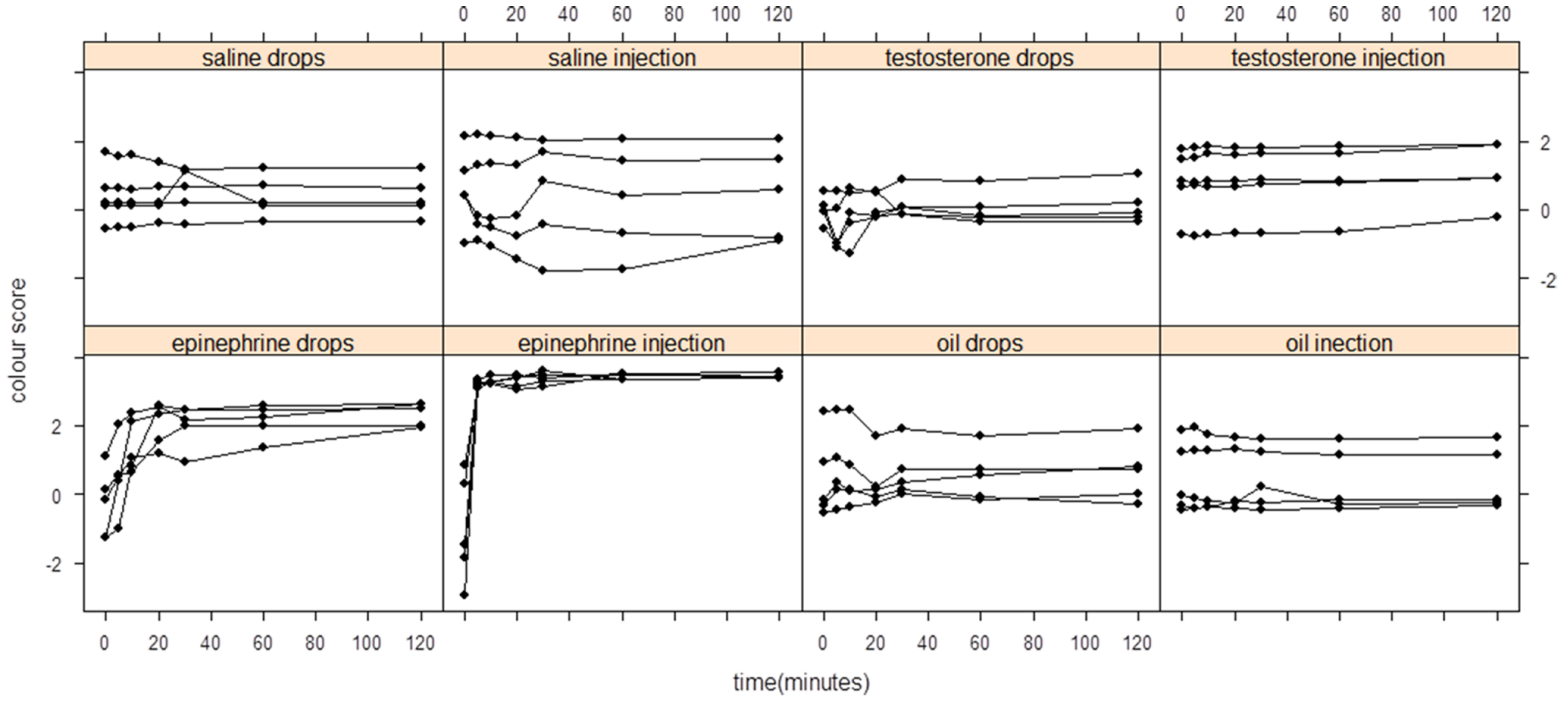
Colour change in *L*. *wilcoxii* in response to control and hormone treatments (n = 5 individual frogs per group). Each point indicates the dorsal colour of a frog at each time point (0–120 minutes).

Baseline colour scores of male frogs at time 0 averaged 0.19 (1.12). There were significant treatment (Fig 3; F = 5.501, df = 7, p = 0.0003) and time effects (Fig 3; F = 26.71, df = 6, p<2e–16) and a significant interaction between treatment and time (Fig 3; F = 14.00, df = 42, p<2e–16). Further post hoc analysis showed that there was a significant difference in dorsal colour following epinephrine injections and topical administration (p<0.001) from 5 to 120 minutes (Video S1). There was no significant difference in dorsal colour scores following testosterone, saline and oil injection or topical administration. Colour scores of epinephrine-injected frogs increased to 3.14 (0.079), similar to the natural colour scores of males recorded in amplexus after 30 minutes (Fig 2). Colour values of frogs subject to non-invasive topical epinephrine administration increased to 2.34 (0.34). There was a significant difference between epinephrine-injected frogs and all other treatments (p = 0.0114). Although topical epinephrine administration induced a similar response (brown to yellow), the magnitude of the response (brightness of the yellow colour) was lower than that following epinephrine injection.

## Discussion

Dynamic colour change occurs in *L. wilcoxii* following the initiation of amplexus. Adult male *L. wilcoxii* change colour from brown or yellow-brown to bright yellow within 5 minutes of amplexing a female and remain yellow for 3–5 hours before fading back to brown. Our natural observations are the first documentation in this species of rapid reversible (brown-yellow-brown) colour change during amplexus. Previously, this type of dynamic colour change phenomenon had only been documented in the Neotropical toad (*Bufo luetkenii*), which fades from a bright yellow to brown following amplexus [10], although the speed of change is slower than that of *L. wilcoxii*. The key difference between this toad and *L. wilcoxii* is that breeding toads change from brown to yellow during the breeding season (prior to amplexus), whereas they undergo a rapid dynamic change from yellow to brown post amplexus.

We replicated this colour change using hormone treatments and identified epinephrine as a likely regulator of rapid colour change in *L. wilcoxii*. While amplexing males faded back to brown over time, this was not observed in experimentally manipulated animals, as frogs were not monitored for long enough. Testosterone injection did not lead to a rapid change colour change. This may have been due the dose injected as previous studies have shown positive results with doses much higher [19]–[21]. These studies used species that are described to change colour at a much slower rate, i.e. over several weeks as in *Hyperolius viridiflavus* and *Acris gryllus* [20], [21] or several days or hours (data not specified) in the *Buergeria robusta* [19]. This may suggest that testosterone works at a slower rate and possibly mediated pigment synthesis rather than rapid pigment aggregation, as observed in *L. wilcoxii*. Due to ethical constraints we were unable to test more doses to investigate this further.

On a cellular level, the colour change in *L. wilcoxii* is likely due to yellow xanthophores (with brightness enhanced by iridophores) being revealed upon aggregation of the melanin in melanophores [14]. Both injections and topical administration of epinephrine induced a dynamic colour change from brown to yellow. We predict that *L. wilcoxii* chromatophores possess receptors for this hormone (α adrenoreceptors), as pigment movement can be directly stimulated this way [37]. It should be noted that, although the topical administration induced a weaker effect, as evidenced by a weaker yellow colour at all time periods after the injection, it provides a reliable and rapid field method for future research into the dynamics of hormonal systems in amphibian colour change (see video S1).

In other animals, the presence of bright signals during pairing often functions as a signal to show dominance over other males [38]–[40]; during our observations we noticed male wilcoxii attempting to displace amplexing males in four occasions. Yellow may therefore be a signal to other males, signalling they have secured a female. Amplexus was rarely interrupted by satellite males although struggles between 2 or more males on one female were observed on some occasions. It should also be noted that female *L. wilcoxii* were not observed to lay egg during the period they were monitored, however, as most females were unable to be found after 6 hours they could have laid eggs after this time.

Alternatively, the bright colouration may have no current adaptive function, and may be a bi-product of hormone release for other purposes, such as amplexus [23], [41]. The hormonal processes of amplexus, specifically hormones triggering sperm release can vary between species, most often human chorionic gonadotropin and luteinizing hormone releasing hormone have been used in captive breeding studies [42], [43]. Previous studies have shown that catecholamines and gonadotropin-releasing hormone-like peptides elicit sperm release in several amphibian species [23], [41], [42], [44] which suggests the possibility that the bright colouration observed in *L. wilcoxii* may be a by-product of initiating sperm release. This may explain the similar responses observed previously where application of a moderate stress (toe-clipping) lead to epinephrine production and therefore colour change [26].

*L. wilcoxii* display an unusual form of colour change, which occurs after initiation of amplexus. This may play an important role in sexual selection as a male-male signal, or it may simply be a nonselective by-product of an essential physiological process, such as initiating sperm release. We have demonstrated that the neuro-hormonal endocrine pathway is likely to be the proximate regulator of this dynamic colour change. In contrast to other colour-changing amphibians, which display bright colours for hours or days prior to amplexus [8], [10], this rapid colour change occurred post-amplexus. Therefore, behavioural studies are needed to understand the evolutionary functions of dynamic colour change. Linking hormonal mechanisms with the adaptive function of this dynamic colour change opens up opportunities for new discoveries into the interactions between physiological processes and amphibian behavioural ecology.

## Supporting Information

Table S1 Individual frog colour score values at for each time point and treatment.
(DOCX)

Video S1 Video showing example rapid colour change in L/wilcoxii in response to epinephrine injection.
(ZIP)
