# Supplementary material for: The Neuro-Hormonal Control of Rapid Dynamic Skin Colour Change in an Amphibian during Amplexus
Source: PLoS One. 2014 Dec 3;9(12):e114120. doi: 10.1371/journal.pone.0114120 (PMC4254939; doi:10.1371/journal.pone.0114120)
Supplement: Table S1 — Individual frog colour score values at for each time point and treatment. (DOCX) [file pone.0114120.s001.docx]

| **frog** | **time** | **colour** | **Treatment** |
| --- | --- | --- | --- |
| 1 | 120 | 3.156 | epinephrine injection |
| 1 | 60 | 3.218 | epinephrine injection |
| 1 | 20 | 3.179 | epinephrine injection |
| 1 | 10 | 3.175 | epinephrine injection |
| 1 | 30 | 3.102 | epinephrine injection |
| 1 | 5 | 3.046 | epinephrine injection |
| 1 | 0 | -1.455 | epinephrine injection |
| 2 | 5 | -0.9 | saline injection |
| 2 | 120 | -0.9 | saline injection |
| 2 | 0 | -0.97 | saline injection |
| 2 | 10 | -1.05 | saline injection |
| 2 | 20 | -1.43 | saline injection |
| 2 | 60 | -1.72 | saline injection |
| 2 | 30 | -1.77 | saline injection |
| 3 | 30 | 3.188 | epinephrine injection |
| 3 | 60 | 3.17 | epinephrine injection |
| 3 | 120 | 3.139 | epinephrine injection |
| 3 | 20 | 3.114 | epinephrine injection |
| 3 | 10 | 2.951 | epinephrine injection |
| 3 | 5 | 2.862 | epinephrine injection |
| 3 | 0 | -2.941 | epinephrine injection |
| 4 | 5 | 2.18 | saline injection |
| 4 | 0 | 2.16 | saline injection |
| 4 | 10 | 2.15 | saline injection |
| 4 | 20 | 2.09 | saline injection |
| 4 | 120 | 2.08 | saline injection |
| 4 | 60 | 2.06 | saline injection |
| 4 | 30 | 2.04 | saline injection |
| 5 | 60 | 3.212 | epinephrine injection |
| 5 | 120 | 3.145 | epinephrine injection |
| 5 | 5 | 2.922 | epinephrine injection |
| 5 | 10 | 2.917 | epinephrine injection |
| 5 | 30 | 2.842 | epinephrine injection |
| 5 | 20 | 2.735 | epinephrine injection |
| 5 | 0 | -1.846 | epinephrine injection |
| 6 | 0 | 0.4 | saline injection |
| 6 | 5 | -0.41 | saline injection |
| 6 | 30 | -0.42 | saline injection |
| 6 | 10 | -0.52 | saline injection |
| 6 | 60 | -0.67 | saline injection |
| 6 | 20 | -0.77 | saline injection |
| 6 | 120 | -0.79 | saline injection |
| 7 | 30 | 3.119 | epinephrine injection |
| 7 | 20 | 3.095 | epinephrine injection |
| 7 | 120 | 3.079 | epinephrine injection |
| 7 | 60 | 3.066 | epinephrine injection |
| 7 | 5 | 2.949 | epinephrine injection |
| 7 | 10 | 2.946 | epinephrine injection |
| 7 | 0 | 0.287 | epinephrine injection |
| 8 | 30 | 1.69 | saline injection |
| 8 | 120 | 1.48 | saline injection |
| 8 | 60 | 1.42 | saline injection |
| 8 | 10 | 1.37 | saline injection |
| 8 | 20 | 1.31 | saline injection |
| 8 | 5 | 1.29 | saline injection |
| 8 | 0 | 1.12 | saline injection |
| 9 | 120 | 3.098 | epinephrine injection |
| 9 | 60 | 3.053 | epinephrine injection |
| 9 | 30 | 3.011 | epinephrine injection |
| 9 | 10 | 2.958 | epinephrine injection |
| 9 | 20 | 2.838 | epinephrine injection |
| 9 | 5 | 2.774 | epinephrine injection |
| 9 | 0 | 0.852 | epinephrine injection |
| 10 | 30 | 0.86 | saline injection |
| 10 | 120 | 0.59 | saline injection |
| 10 | 60 | 0.43 | saline injection |
| 10 | 0 | 0.42 | saline injection |
| 10 | 20 | -0.15 | saline injection |
| 10 | 5 | -0.19 | saline injection |
| 10 | 10 | -0.24 | saline injection |
| 11 | 120 | 2.491 | epinephrine drops |
| 11 | 60 | 2.471 | epinephrine drops |
| 11 | 30 | 2.441 | epinephrine drops |
| 11 | 20 | 2.314 | epinephrine drops |
| 11 | 10 | 2.106 | epinephrine drops |
| 11 | 5 | 0.385 | epinephrine drops |
| 11 | 0 | -1.246 | epinephrine drops |
| 12 | 120 | 2.001 | epinephrine drops |
| 12 | 60 | 1.999 | epinephrine drops |
| 12 | 30 | 1.994 | epinephrine drops |
| 12 | 20 | 1.594 | epinephrine drops |
| 12 | 10 | 0.659 | epinephrine drops |
| 12 | 5 | 0.551 | epinephrine drops |
| 12 | 0 | 0.128 | epinephrine drops |
| 13 | 120 | 2.611 | epinephrine drops |
| 13 | 20 | 2.56 | epinephrine drops |
| 13 | 10 | 2.368 | epinephrine drops |
| 13 | 60 | 2.241 | epinephrine drops |
| 13 | 30 | 2.174 | epinephrine drops |
| 13 | 5 | 2.02 | epinephrine drops |
| 13 | 0 | 1.091 | epinephrine drops |
| 14 | 120 | 2.636 | epinephrine drops |
| 14 | 60 | 2.606 | epinephrine drops |
| 14 | 20 | 2.573 | epinephrine drops |
| 14 | 30 | 2.48 | epinephrine drops |
| 14 | 10 | 0.834 | epinephrine drops |
| 14 | 5 | -1.015 | epinephrine drops |
| 14 | 0 | -1.254 | epinephrine drops |
| 15 | 120 | 1.936 | epinephrine drops |
| 15 | 60 | 1.352 | epinephrine drops |
| 15 | 20 | 1.187 | epinephrine drops |
| 15 | 10 | 1.067 | epinephrine drops |
| 15 | 30 | 0.929 | epinephrine drops |
| 15 | 5 | 0.535 | epinephrine drops |
| 15 | 0 | -0.154 | epinephrine drops |
| 16 | 0 | -0.05 | testosterone drops |
| 16 | 5 | 0.05 | testosterone drops |
| 16 | 10 | 0.62 | testosterone drops |
| 16 | 20 | 0.5 | testosterone drops |
| 16 | 30 | 0.87 | testosterone drops |
| 16 | 60 | 0.85 | testosterone drops |
| 16 | 120 | 1.07 | testosterone drops |
| 17 | 0 | 0.11 | testosterone drops |
| 17 | 5 | -0.96 | testosterone drops |
| 17 | 10 | -0.4 | testosterone drops |
| 17 | 20 | -0.2 | testosterone drops |
| 17 | 30 | 0.07 | testosterone drops |
| 17 | 60 | 0.09 | testosterone drops |
| 17 | 120 | 0.19 | testosterone drops |
| 18 | 0 | -0.54 | testosterone drops |
| 18 | 5 | -0.96 | testosterone drops |
| 18 | 10 | -0.09 | testosterone drops |
| 18 | 20 | -0.19 | testosterone drops |
| 18 | 30 | -0.13 | testosterone drops |
| 18 | 60 | -0.22 | testosterone drops |
| 18 | 120 | -0.22 | testosterone drops |
| 19 | 0 | 0.55 | testosterone drops |
| 19 | 5 | 0.56 | testosterone drops |
| 19 | 10 | 0.49 | testosterone drops |
| 19 | 20 | 0.55 | testosterone drops |
| 19 | 30 | -0.13 | testosterone drops |
| 19 | 60 | -0.35 | testosterone drops |
| 19 | 120 | -0.35 | testosterone drops |
| 20 | 0 | -0.03 | testosterone drops |
| 20 | 5 | -1.12 | testosterone drops |
| 20 | 10 | -1.26 | testosterone drops |
| 20 | 20 | -0.1 | testosterone drops |
| 20 | 30 | 0.1 | testosterone drops |
| 20 | 60 | -0.15 | testosterone drops |
| 20 | 120 | -0.09 | testosterone drops |
| 21 | 0 | 0.85 | testosterone injection |
| 21 | 5 | 0.82 | testosterone injection |
| 21 | 10 | 0.83 | testosterone injection |
| 21 | 20 | 0.86 | testosterone injection |
| 21 | 30 | 0.87 | testosterone injection |
| 21 | 60 | 0.84 | testosterone injection |
| 21 | 120 | 0.91 | testosterone injection |
| 22 | 0 | -0.44 | oil inection |
| 22 | 5 | -0.39 | oil inection |
| 22 | 10 | -0.38 | oil inection |
| 22 | 20 | -0.43 | oil inection |
| 22 | 30 | -0.46 | oil inection |
| 22 | 60 | -0.43 | oil inection |
| 22 | 120 | -0.31 | oil inection |
| 23 | 0 | 1.79 | testosterone injection |
| 23 | 5 | 1.81 | testosterone injection |
| 23 | 10 | 1.85 | testosterone injection |
| 23 | 20 | 1.82 | testosterone injection |
| 23 | 30 | 1.83 | testosterone injection |
| 23 | 60 | 1.87 | testosterone injection |
| 23 | 120 | 1.91 | testosterone injection |
| 24 | 0 | 1.89 | oil inection |
| 24 | 5 | 1.95 | oil inection |
| 24 | 10 | 1.73 | oil inection |
| 24 | 20 | 1.68 | oil inection |
| 24 | 30 | 1.63 | oil inection |
| 24 | 60 | 1.61 | oil inection |
| 24 | 120 | 1.68 | oil inection |
| 25 | 0 | 0.69 | testosterone injection |
| 25 | 5 | 0.71 | testosterone injection |
| 25 | 10 | 0.68 | testosterone injection |
| 25 | 20 | 0.66 | testosterone injection |
| 25 | 30 | 0.77 | testosterone injection |
| 25 | 60 | 0.81 | testosterone injection |
| 25 | 120 | 0.92 | testosterone injection |
| 26 | 0 | 0.96 | oil drops |
| 26 | 5 | 1.05 | oil drops |
| 26 | 10 | 0.87 | oil drops |
| 26 | 20 | 0.22 | oil drops |
| 26 | 30 | 0.73 | oil drops |
| 26 | 60 | 0.73 | oil drops |
| 26 | 120 | 0.73 | oil drops |
| 27 | 0 | -0.16 | oil drops |
| 27 | 5 | 0.35 | oil drops |
| 27 | 10 | 0.08 | oil drops |
| 27 | 20 | 0.13 | oil drops |
| 27 | 30 | 0.36 | oil drops |
| 27 | 60 | 0.58 | oil drops |
| 27 | 120 | 0.81 | oil drops |
| 28 | 0 | -0.34 | oil drops |
| 28 | 5 | 0.16 | oil drops |
| 28 | 10 | 0.14 | oil drops |
| 28 | 20 | -0.08 | oil drops |
| 28 | 30 | 0.14 | oil drops |
| 28 | 60 | -0.08 | oil drops |
| 28 | 120 | -0.29 | oil drops |
| 29 | 60 | 0.7 | saline drops |
| 29 | 20 | 0.67 | saline drops |
| 29 | 30 | 0.66 | saline drops |
| 29 | 5 | 0.65 | saline drops |
| 29 | 0 | 0.63 | saline drops |
| 29 | 120 | 0.62 | saline drops |
| 29 | 10 | 0.6 | saline drops |
| 30 | 60 | -0.32 | saline drops |
| 30 | 120 | -0.33 | saline drops |
| 30 | 20 | -0.38 | saline drops |
| 30 | 30 | -0.41 | saline drops |
| 30 | 5 | -0.49 | saline drops |
| 30 | 10 | -0.51 | saline drops |
| 30 | 0 | -0.53 | saline drops |
| 31 | 0 | -0.31 | oil inection |
| 31 | 5 | -0.42 | oil inection |
| 31 | 10 | -0.36 | oil inection |
| 31 | 20 | -0.19 | oil inection |
| 31 | 30 | -0.22 | oil inection |
| 31 | 60 | -0.16 | oil inection |
| 31 | 120 | -0.15 | oil inection |
| 32 | 0 | 1.245 | oil inection |
| 32 | 5 | 1.28 | oil inection |
| 32 | 10 | 1.26 | oil inection |
| 32 | 20 | 1.31 | oil inection |
| 32 | 30 | 1.24 | oil inection |
| 32 | 60 | 1.16 | oil inection |
| 32 | 120 | 1.14 | oil inection |
| 33 | 0 | -0.04 | oil inection |
| 33 | 5 | -0.12 | oil inection |
| 33 | 10 | -0.18 | oil inection |
| 33 | 20 | -0.25 | oil inection |
| 33 | 30 | 0.22 | oil inection |
| 33 | 60 | -0.28 | oil inection |
| 33 | 120 | -0.25 | oil inection |
| 34 | 0 | 2.43 | oil drops |
| 34 | 5 | 2.44 | oil drops |
| 34 | 10 | 2.47 | oil drops |
| 34 | 20 | 1.71 | oil drops |
| 34 | 30 | 1.9 | oil drops |
| 34 | 60 | 1.71 | oil drops |
| 34 | 120 | 1.9 | oil drops |
| 35 | 0 | -0.55 | oil drops |
| 35 | 5 | -0.44 | oil drops |
| 35 | 10 | -0.38 | oil drops |
| 35 | 20 | -0.26 | oil drops |
| 35 | 30 | 0.02 | oil drops |
| 35 | 60 | -0.16 | oil drops |
| 35 | 120 | 0.02 | oil drops |
| 36 | 0 | 1.47 | testosterone injection |
| 36 | 5 | 1.53 | testosterone injection |
| 36 | 10 | 1.65 | testosterone injection |
| 36 | 20 | 1.61 | testosterone injection |
| 36 | 30 | 1.64 | testosterone injection |
| 36 | 60 | 1.651 | testosterone injection |
| 36 | 120 | 1.895 | testosterone injection |
| 37 | 0 | -0.74 | testosterone injection |
| 37 | 5 | -0.75 | testosterone injection |
| 37 | 10 | -0.73 | testosterone injection |
| 37 | 20 | -0.68 | testosterone injection |
| 37 | 30 | -0.67 | testosterone injection |
| 37 | 60 | -0.63 | testosterone injection |
| 37 | 120 | -0.2 | testosterone injection |
| 38 | 0 | 1.68 | saline drops |
| 38 | 10 | 1.61 | saline drops |
| 38 | 5 | 1.58 | saline drops |
| 38 | 20 | 1.38 | saline drops |
| 38 | 120 | 1.23 | saline drops |
| 38 | 60 | 1.21 | saline drops |
| 38 | 30 | 1.19 | saline drops |
| 39 | 30 | 1.136 | saline drops |
| 39 | 0 | 0.14 | saline drops |
| 39 | 20 | 0.137 | saline drops |
| 39 | 10 | 0.136 | saline drops |
| 39 | 60 | 0.132 | saline drops |
| 39 | 120 | 0.131 | saline drops |
| 39 | 5 | 0.13 | saline drops |
| 40 | 10 | 0.21 | saline drops |
| 40 | 60 | 0.208 | saline drops |
| 40 | 120 | 0.197 | saline drops |
| 40 | 5 | 0.195 | saline drops |
| 40 | 20 | 0.192 | saline drops |
| 40 | 0 | 0.19 | saline drops |
| 40 | 30 | 0.19 | saline drops |
| 41 | 0 | -1.279 | natural |
| 42 | 0 | -0.128 | natural |
| 43 | 0 | 2.85 | natural |
| 44 | 0 | -2.239 | natural |
| 45 | 0 | -2.011 | female |
| 46 | 0 | -1.19 | natural |
| 47 | 0 | -1.104 | natural |
| 48 | 0 | 1.404 | natural |
| 49 | 0 | 2.264 | natural |
| 50 | 0 | 1.917 | natural |
| 51 | 0 | 1.917 | natural |
| 52 | 0 | 1.917 | natural |
| 53 | 0 | -1.074 | natural |
| 54 | 0 | 1.343 | natural |
| 55 | 0 | 2.204 | natural |
| 56 | 0 | 2.064 | natural |
| 57 | 0 | -1.53 | natural |
| 58 | 0 | -1.917 | natural |
| 59 | 0 | -1.582 | natural |
| 41 | 5 | 2.665 | natural |
| 42 | 5 | 2.67 | natural |
| 43 | 5 | 2.926 | natural |
| 44 | 5 | 2.397 | natural |
| 45 | 5 | -2.204 | female |
| 46 | 5 | 2.378 | natural |
| 47 | 5 | 2.746 | natural |
| 48 | 5 | 2.404 | natural |
| 49 | 5 | 3.17 | natural |
| 50 | 5 | 2.917 | natural |
| 51 | 5 | 2.4 | natural |
| 52 | 5 | 2.27 | natural |
| 53 | 5 | 2.22 | natural |
| 54 | 5 | 2.546 | natural |
| 55 | 5 | 2.204 | natural |
| 56 | 5 | 2.33 | natural |
| 57 | 5 | 2.148 | natural |
| 58 | 5 | 1.986 | natural |
| 59 | 5 | 2.057 | natural |
| 41 | 10 | 2.498 | natural |
| 42 | 10 | 3.538 | natural |
| 43 | 10 | 2.924 | natural |
| 44 | 10 | 2.454 | natural |
| 45 | 10 | -2.235 | female |
| 46 | 10 | 2.378 | natural |
| 47 | 10 | 2.746 | natural |
| 48 | 10 | 2.404 | natural |
| 49 | 10 | 3.073 | natural |
| 50 | 10 | 2.917 | natural |
| 51 | 10 | 2.4 | natural |
| 52 | 10 | 2.217 | natural |
| 53 | 10 | 2.474 | natural |
| 54 | 10 | 2.548 | natural |
| 55 | 10 | 2.304 | natural |
| 56 | 10 | 2.33 | natural |
| 57 | 10 | 2.169 | natural |
| 58 | 10 | 1.682 | natural |
| 59 | 10 | 2.218 | natural |
| 41 | 20 | 2.641 | natural |
| 42 | 20 | 3.239 | natural |
| 43 | 20 | 2.948 | natural |
| 44 | 20 | 2.454 | natural |
| 45 | 20 | -2.228 | female |
| 46 | 20 | 2.378 | natural |
| 47 | 20 | 2.746 | natural |
| 48 | 20 | 2.345 | natural |
| 49 | 20 | 3.103 | natural |
| 50 | 20 | 2.917 | natural |
| 51 | 20 | 2.4 | natural |
| 52 | 20 | 2.417 | natural |
| 53 | 20 | 2.615 | natural |
| 54 | 20 | 2.486 | natural |
| 55 | 20 | 2.204 | natural |
| 56 | 20 | 2.33 | natural |
| 57 | 20 | 2.327 | natural |
| 58 | 20 | 1.986 | natural |
| 59 | 20 | 2.416 | natural |
| 41 | 30 | 2.653 | natural |
| 42 | 30 | 3.533 | natural |
| 43 | 30 | 2.763 | natural |
| 44 | 30 | 2.852 | natural |
| 45 | 30 | -2.203 | female |
| 46 | 30 | 2.383 | natural |
| 47 | 30 | 2.746 | natural |
| 48 | 30 | 2.315 | natural |
| 49 | 30 | 3.103 | natural |
| 50 | 30 | 2.8 | natural |
| 51 | 30 | 2.4 | natural |
| 52 | 30 | 2.3 | natural |
| 53 | 30 | 2.663 | natural |
| 54 | 30 | 2.346 | natural |
| 55 | 30 | 3.015 | natural |
| 56 | 30 | 2.23 | natural |
| 57 | 30 | 2.863 | natural |
| 58 | 30 | 2.393 | natural |
| 59 | 30 | 2.414 | natural |
| 42 | 60 | 2.77 | natural |
| 43 | 60 | 2.82 | natural |
| 44 | 60 | 2.847 | natural |
| 45 | 60 | -2.235 | female |
| 46 | 60 | 2.478 | natural |
| 47 | 60 | 2.746 | natural |
| 48 | 60 | 2.284 | natural |
| 49 | 60 | 3.106 | natural |
| 50 | 60 | 2.83 | natural |
| 51 | 60 | 2.4 | natural |
| 52 | 60 | 2.4 | natural |
| 53 | 60 | 2.4 | natural |
| 54 | 60 | 2.546 | natural |
| 55 | 60 | 2.769 | natural |
| 56 | 60 | 2.23 | natural |
| 57 | 60 | 2.863 | natural |
| 58 | 60 | 2.4 | natural |
| 59 | 60 | 2.391 | natural |
| 42 | 90 | 2.789 | natural |
| 43 | 90 | 2.47 | natural |
| 44 | 90 | 2.84 | natural |
| 45 | 90 | -2.086 | female |
| 46 | 90 | 2.395 | natural |
| 47 | 90 | 2.746 | natural |
| 48 | 90 | 2.284 | natural |
| 49 | 90 | 3.103 | natural |
| 50 | 90 | 2.8 | natural |
| 51 | 90 | 2.4 | natural |
| 52 | 90 | 2.563 | natural |
| 53 | 90 | 2.4 | natural |
| 54 | 90 | 2.476 | natural |
| 55 | 90 | 2.769 | natural |
| 56 | 90 | 2.33 | natural |
| 57 | 90 | 2.863 | natural |
| 58 | 90 | 2.447 | natural |
| 59 | 90 | 2.37 | natural |
| 42 | 120 | 2.695 | natural |
| 43 | 120 | 2.462 | natural |
| 44 | 120 | 2.818 | natural |
| 45 | 120 | -2.207 | female |
| 46 | 120 | 2.578 | natural |
| 47 | 120 | 2.746 | natural |
| 48 | 120 | 2.284 | natural |
| 49 | 120 | 3.103 | natural |
| 50 | 120 | 2.8 | natural |
| 51 | 120 | 2.4 | natural |
| 52 | 120 | 2.4 | natural |
| 53 | 120 | 2.563 | natural |
| 54 | 120 | 2.483 | natural |
| 55 | 120 | 2.769 | natural |
| 56 | 120 | 2.33 | natural |
| 57 | 120 | 2.563 | natural |
| 58 | 120 | 2.391 | natural |
| 59 | 120 | 2.379 | natural |
| 42 | 150 | 2.732 | natural |
| 43 | 150 | 2.423 | natural |
| 44 | 150 | 2.735 | natural |
| 45 | 150 | -2.011 | female |
| 46 | 150 | 2.478 | natural |
| 47 | 150 | 2.746 | natural |
| 48 | 150 | 2.284 | natural |
| 49 | 150 | 3.103 | natural |
| 50 | 150 | 2.8 | natural |
| 51 | 150 | 2.4 | natural |
| 52 | 150 | 2.4 | natural |
| 53 | 150 | 2.4 | natural |
| 54 | 150 | 2.646 | natural |
| 55 | 150 | 2.729 | natural |
| 56 | 150 | 2.33 | natural |
| 57 | 150 | 2.709 | natural |
| 58 | 150 | 2.4 | natural |
| 59 | 150 | 2.415 | natural |
| 42 | 180 | 3.301 | natural |
| 43 | 180 | 2.456 | natural |
| 44 | 180 | 3.008 | natural |
| 45 | 180 | -2.104 | female |
| 46 | 180 | 2.595 | natural |
| 47 | 180 | 2.746 | natural |
| 48 | 180 | 2.284 | natural |
| 49 | 180 | 3.103 | natural |
| 50 | 180 | 2.3 | natural |
| 51 | 180 | 2.4 | natural |
| 52 | 180 | 2.3 | natural |
| 53 | 180 | 2.506 | natural |
| 54 | 180 | 2.746 | natural |
| 55 | 180 | 2.769 | natural |
| 56 | 180 | 2.33 | natural |
| 57 | 180 | 2.473 | natural |
| 58 | 180 | 2.37 | natural |
| 59 | 180 | 2.403 | natural |
| 44 | 210 | 2.734 | natural |
| 45 | 210 | -2.135 | female |
| 46 | 210 | -0.648 | natural |
| 47 | 210 | 2.746 | natural |
| 48 | 210 | 2.276 | natural |
| 49 | 210 | 3.103 | natural |
| 50 | 210 | 2.2 | natural |
| 51 | 210 | 2.4 | natural |
| 52 | 210 | 2.4 | natural |
| 53 | 210 | 2.4 | natural |
| 54 | 210 | 2.546 | natural |
| 55 | 210 | 2.061 | natural |
| 56 | 210 | -1.873 | natural |
| 57 | 210 | 2.6 | natural |
| 58 | 210 | 2.363 | natural |
| 59 | 210 | 2.4 | natural |
| 42 | 240 | -1.066 | natural |
| 44 | 240 | -1.233 | natural |
| 45 | 240 | -2.128 | female |
| 46 | 240 | -1.096 | natural |
| 47 | 240 | 2.746 | natural |
| 48 | 240 | 1.023 | natural |
| 49 | 240 | 2.073 | natural |
| 50 | 240 | 1.936 | natural |
| 51 | 240 | 1.936 | natural |
| 52 | 240 | 1.936 | natural |
| 53 | 240 | 1.936 | natural |
| 54 | 240 | 2.533 | natural |
| 55 | 240 | 0.823 | natural |
| 56 | 240 | -2.217 | natural |
| 57 | 240 | 1.836 | natural |
| 58 | 240 | 1.936 | natural |
| 59 | 240 | 1.936 | natural |
| 45 | 270 | -2.111 | female |
| 47 | 270 | 2.746 | natural |
| 48 | 270 | 0.986 | natural |
| 50 | 270 | -0.778 | natural |
| 51 | 270 | -1.078 | natural |
| 52 | 270 | -1.078 | natural |
| 53 | 270 | -2.078 | natural |
| 54 | 270 | 2.046 | natural |
| 55 | 270 | -2.786 | natural |
| 57 | 270 | -1.064 | natural |
| 58 | 270 | -1.157 | natural |
| 59 | 270 | -1.078 | natural |
| 45 | 300 | -2.094 | female |
| 47 | 300 | 2.746 | natural |
| 50 | 300 | -1.078 | natural |
| 51 | 300 | -1.078 | natural |
| 52 | 300 | -1.078 | natural |
| 53 | 300 | -2.585 | natural |
| 54 | 300 | -0.606 | natural |
| 57 | 300 | -1.379 | natural |
| 58 | 300 | -1.276 | natural |
| 59 | 300 | -1.078 | natural |
| 47 | 330 | -0.878 | natural |
| 54 | 330 | -1.178 | natural |
